# Case Report: A novel *RRM2B* variant in a Chinese infant with mitochondrial DNA depletion syndrome and collective analyses of *RRM2B* variants for disease etiology

**DOI:** 10.3389/fped.2024.1363728

**Published:** 2024-04-25

**Authors:** Yanjun Wang, Ling Hang, Weihua Shou, Cuifen Li, Fangling Dong, Xingxing Feng, Ruohong Jin, Bin Li, Shufang Xiao

**Affiliations:** ^1^ Pediatric Intensive Care Unit, Kunming Children’s Hospital, Children’s Hospital Affiliated to Kunming Medical University, Kunming, China; ^2^ Kunming Key Laboratory of Children Infection and Immunity, Yunnan Key Laboratory of Children’s Major Disease Research, Yunnan Medical Center for Pediatric Diseases, Yunnan Institute of Pediatrics, Kunming Children’s Hospital, Kunming, China; ^3^ Department of Clinical Laboratory, Kunming Children’s Hospital, Children’s Hospital Affiliated to Kunming Medical University, Kunming, China

**Keywords:** mitochondrial DNA depletion syndrome, *RRM2B*, whole-exome sequencing, disease spectrum, disease etiology

## Abstract

**Background:**

There are few reports of infantile mitochondrial DNA depletion syndrome (MDDS) caused by variants in *RRM2B* and the correlation between genotype and phenotype has rarely been analyzed in detail. This study investigated an infantile patient with MDDS, from clinical characteristics to genetic causes.

**Methods:**

Routine physical examinations, laboratory assays, which included gas chromatography–mass spectrometry of blood and urine, and MRI scans were performed to obtain an exact diagnosis. Whole-exome sequencing was used to pinpoint the abnormal gene and bioinformatic analyses were performed on the identified variant.

**Results:**

The case presented with progressive neurologic deterioration, failure to thrive, respiratory distress and lactic acidosis. Sequencing revealed that the patient had a homozygous novel missense variant, c.155T>C (p.Ile52Thr), in exon 2 of the *RRM2B* gene. Multiple lines of bioinformatic evidence suggested that this was a likely detrimental variant. In addition, reported *RRM2B* variants were compiled from the relevant literature to analyze disease etiology. We found a distinctive distribution of genotypes across disease manifestations of different severity. Pathogenic alleles of *RRM2B* were significantly enriched in MDDS cases.

**Conclusion:**

The novel variant is a likely genetic cause of MDDS. It expands our understanding of the pathogenic variant spectrum and the contribution of the *RRM2B* gene to the disease spectrum of MDDS.

## 1 Introduction

**Figure 1:**
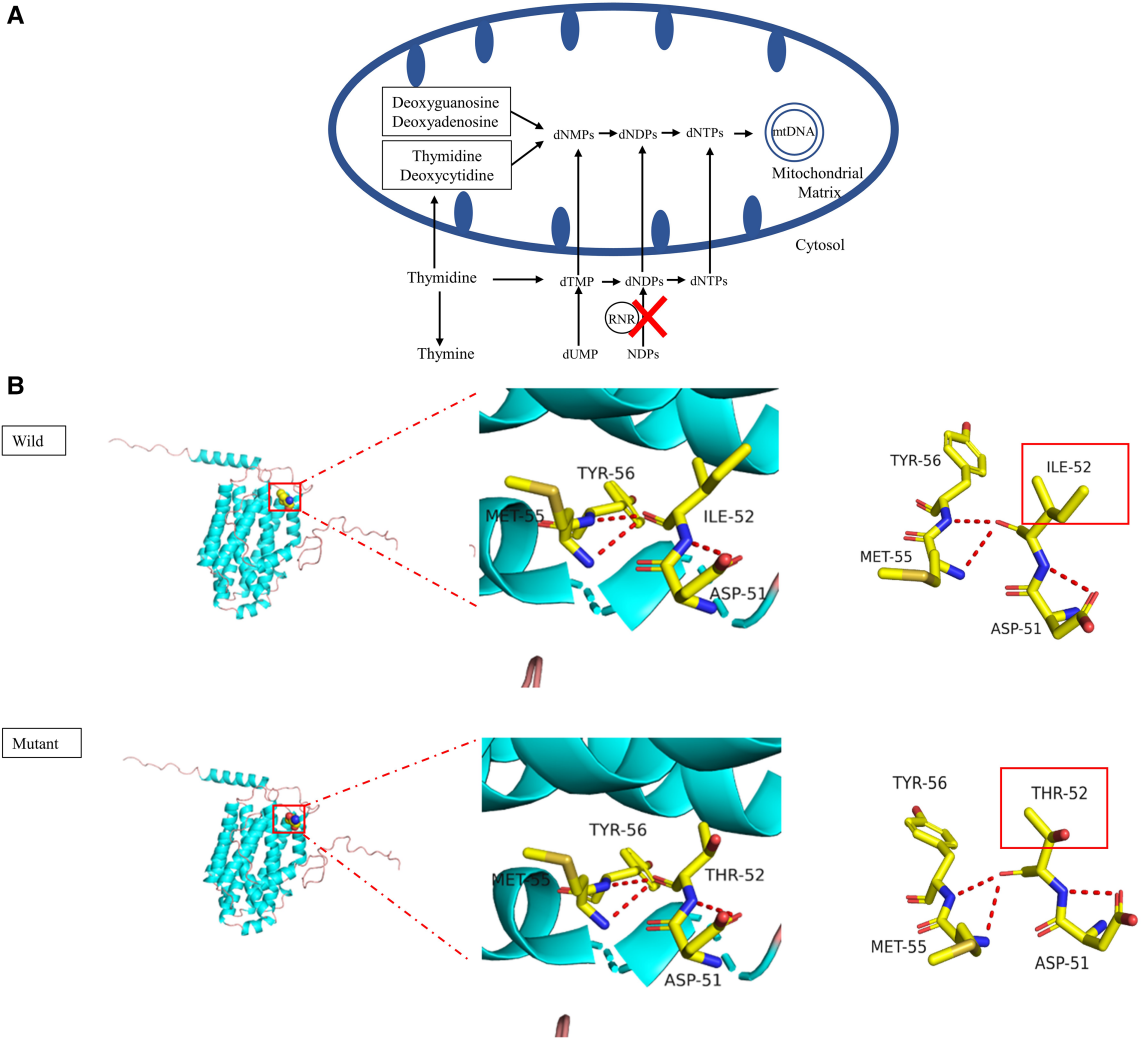
(**A**) schematic presentation of mtDNA replication. RNR, ribonucleotide reductase; dNMP, deoxynucleoside monophosphate; dNDP, deoxynucleoside diphosphate; dNTP, deoxynucleoside triphosphate; NDP, nucleoside diphosphate; dTMP, deoxythymidine monophosphate. (**B**) Graphical representation of the RRM2B protein 3D structure and location of the altered amino acid. The identified c. 155T>C variant caused the p.Ile52Thr amino acid change. The pink coil represents the loop structure, blue depicts an alpha-helix and the purple region is a β-strand. Each color represents a different atom; yellow-C atom, gray-H atom, blue-N atom, red-O atom and orange-S atom. The red dotted line represents the hydrogen bond. The red rectangles show areas of change.

Mitochondrial DNA (mtDNA) depletion syndrome (MDDS), which features a maintenance defect of mtDNA, belongs to a group of disorders with complex phenotypic subtypes due to disrupted nuclear genes (1, 2). Such nuclear genes responsible for MDDS act through the key enzymes that affect the process of mtDNA synthesis and replication, causing a reduction in the amount of mtDNA. This damages the synthesis of the respiratory chain complex and adenosine triphosphate (ATP) in cells, which results in dysfunction in affected tissues and organs. A growing number of studies have expanded the number of pathogenic variants in different genes found in different MDDS phenotypes. They have also established that pathogenic variants in the *RRM2B* gene are an uncommon cause of MDDS, specifically MDDS types 8A and 8B (MIM #612075) (3, 4). The *RRM2B* gene is located on chromosome 8q22 and encodes p53R2, which is a subunit of the ribonucleotide reductase (RNR) complex (5). This complex is a heterotetrametric enzyme and is a key protein in the *de novo* synthesis of mtDNA (Figure 1A). *RRM2B* variants have been found to induce a broad spectrum of clinical phenotypes, from birth to late adulthood, with different inheritance models (6), such as infantile-onset MDDS by autosomal recessive inheritance (7–12), progressive external ophthalmoplegia (PEO) by either autosomal recessive inheritance or autosomal dominant inheritance (7, 13–16) and acute liver failure (17).

To date, fewer than 40 cases of infantile-onset MDDS caused by an *RRM2B* defect have been reported (9, 18–21). These cases mainly presented with hypotonia, progressive neurologic deterioration, failure to thrive, lactic acidosis, feeding difficulties, respiratory distress and proximal tubulopathy. Other symptoms, such as sensorineural hearing loss (SNHL), gastrointestinal (GI) symptoms and seizures, also emerged in several infants. The patients were usually hospitalized due to hypotonia, feeding difficulties and failure to thrive in the first few months after birth. Laboratory tests showed elevated lactate levels in multiple body fluids, such as blood, cerebrospinal fluid (CSF) and urine, deficiency in oxidative phosphorylation enzymes and loss of residual mtDNA in skeletal muscle (8). The patients usually had a low overall survival rate, which dropped to 29% and 16% at 6 months and 1 year after birth, respectively (18).

In this study, we characterized the clinical features of an infantile patient with fatal MDDS from a Chinese Han trio family. The patient was identified as being homozygous for a novel missense *RRM2B* variant that has not previously been recorded in any of the databases. We analyzed data from a systemic examination and metabolite screen, plus the bioinformatic analyses and protein structure models of the variant, to substantiate the genotype-phenotype correlation in this MDDS case. In addition, we compiled data from the reported *RRM2B* variants and detailed the history of the corresponding cases from the relevant literature in an effort to explore the genetic pathogenicity of these variants. This work will be helpful in extending our understanding of the genetics that underpin MDDS.

## 2 Case report

### 2.1 Clinical features and laboratory assays

The patient was a boy who was 2 months and 12-days-old. He was the first child of non-consanguineous Chinese Han parents, neither of whom had any obvious relevant symptoms or family history of similar diseases. The patient was born at term, after a normal pregnancy, and the birth weight was 3,400 g. The boy was hospitalized in the maternity hospital for 9 days due to asphyxia at birth. Two months later, he was transferred to our hospital due to feeding difficulties and respiratory distress. He then underwent a detailed clinical assessment and laboratory examination.

**Figure 2:**
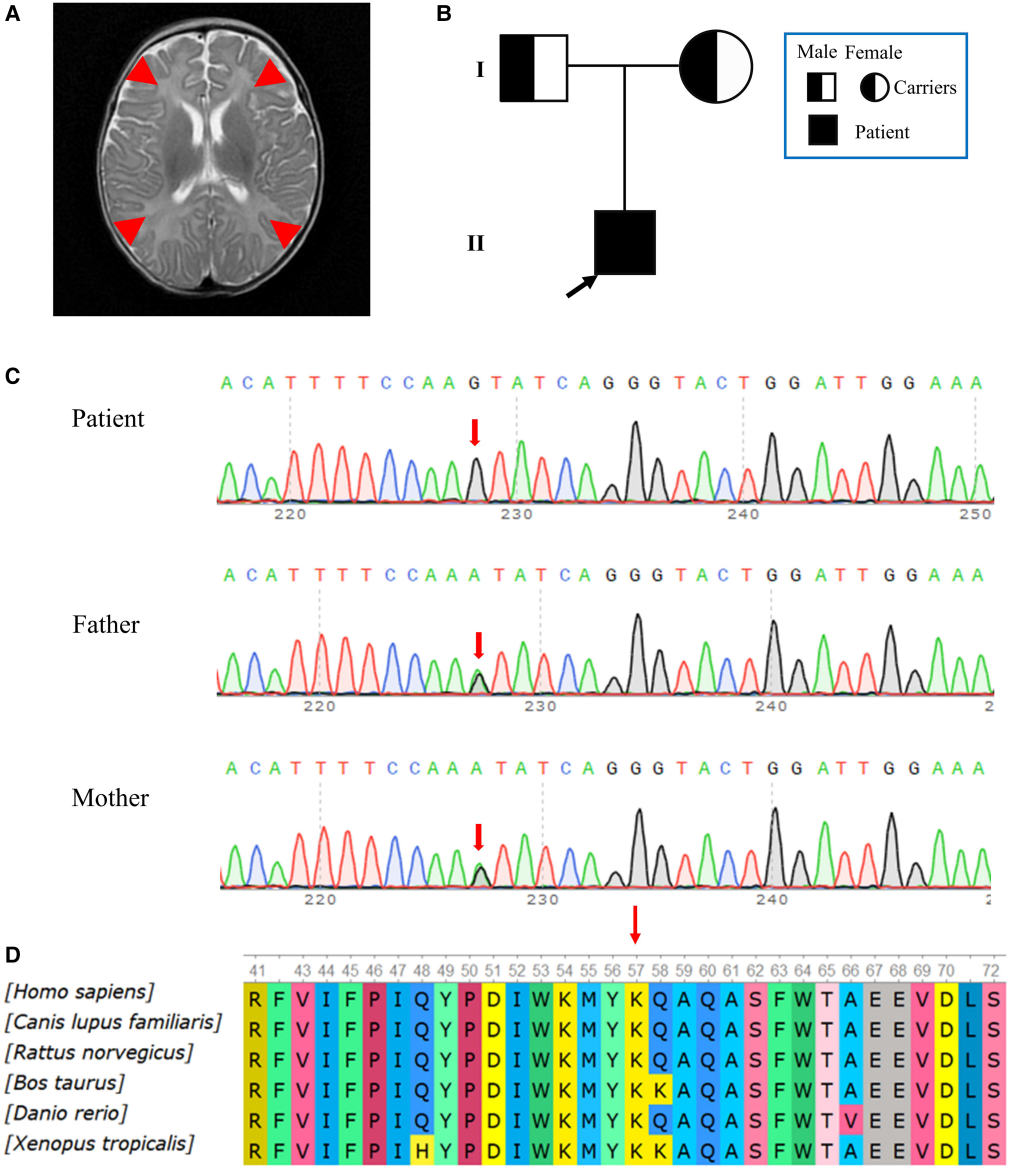
(**A**) MRI images obtained during hospitalization. The MRI showed a high water content in the white matter of the bilateral frontal, temporal, parietal and occipital lobes. Red arrows point to brain lesions. (**B**) Pedigree of the family. The father and mother were asymptomatic carriers of the pathogenic variant, while the patient showed clinical symptoms. (**C**) Reverse complementary sequences of the *RRM2B* variants showed an A>G nucleotide substitution at the 155th nucleotide, in exon 2. The patient carried a homozygous mutation, while both his father and mother had a heterozygous mutation at the same site. (**D**) A multispecies analysis showed that this site was highly conserved.

The patient showed progressive neurologic deterioration and failure to thrive, such as poor suck, lack of head control, hypotonia and lethargy. A progressive dyspnea also emerged during his hospitalization. The hematological parameters (Table 1) showed that the patient had aberrantly elevated lactate (Supplementary Figure S1), high-sensitivity cardiac troponin I (hs-cTnI), myohemoglobin, lactate dehydrogenase (LDH), creatine kinase (CK), aspartic transaminase (AST), alanine aminotransaminase (ALT), plus reduced uric acid and fibrinogen. The serum ammonia level (16.5 umol/L, normal range: 10–50 umol/L) and blood glucose level (4.8 mmol/L, normal range: 3.9–5.8 mmol/L) were normal, whilst the total protein (CSF-TP) level was elevated in the CSF. The results of the patient's electroencephalogram were normal. Multiple pathogens were detected in the patient, such as extended-spectrum β-lactamase-producing *Escherichia coli*, *Streptococcus pneumoniae* and *Pneumocystis jiroveci*. Cardiac ultrasonography indicated pulmonary arterial hypertension, with a 50 mmHg pulmonary arterial systolic pressure and patent foramen ovale. The qualitative test for urinary protein was positive. The MRI showed a high water content in the white matter of the bilateral frontal, temporal, parietal and occipital lobes, and showed that the sulci of the bilateral cerebral hemispheres were wider and deeper than normal, which suggested abnormal brain development (Figure 2A). Urine gas chromatography-mass spectrometry (GC-MS) showed that a variety of urinary organic acids, such as lactate, pyruvic acid, hydroxybutyrate and 5-oxyproline, were elevated by varying degrees (Supplementary Figure S2). As the disease had an impact on mitochondrial function and infection further decreased the ability of the liver cells to metabolize amino acids, an increase in amino acids was observed in the blood. As shown in Table 1, tandem mass spectrometry of blood slides showed that the levels of glutamic acid, glycine ornithine and serine were increased. The levels of acylcarnitine were normal. These results suggested disorders of mitochondrial energy metabolism.

**Table 1:** Hematological parameters of patient.

Items	Reference range	Results	Trend
CSF-TP	0–0.4 g/L	1.94	↑
hs-cTnI	12.7–24.9 pg/ml	137.6	↑
Myohemoglobin	23–72 ng/ml	118.5	↑
LDH	67–394.1 U/L	970	↑
CK	16.5–211.5 U/L	499	↑
AST	0–40 U/L	122	↑
ALT	0–40 U/L	80	↑
Uric acid	119–327 umol/L	60	↓
Fibrinogen	2–4 g/L	0.87	↓
Glutamic acid	45–200 umol/L	276.257	↑
Glycine	90–350 umol/L	429.094	↑
Ornithine	15–80 umol/L	101.846	↑
Serine	20–100 umol/L	163.261	↑
Arg/Orn	0.05–0.7 umol/L	0.024	↓
Orn/Cit	0.8–4 umol/L	5.133	↑

CSF-TP, cerebrospinal fluid total protein; hs-cTnI, high-sensitivity cardiac troponin I; LDH, lactate dehydrogenase; CK, creatine kinase; AST, aspartic transaminase; ALT, alanine aminotransaminase.

Due to failure to cure the patient's progressive dyspnea with 8 days of high-flow nasal cannula oxygen therapy, the patient was treated with oral tracheal intubation and mechanical ventilation for 30 days. The patient's condition was complicated by multiple pathogenic infections, which may have resulted in liver and myocardial damage. The patient received comprehensive treatments, such as meropenem, vancomycin, caspofungin and sulfonamide for infection, inflammation, immune regulation, myocardial nutrition, correction of acidosis and maintenance of internal environment stability. The patient could not be weaned-off the ventilator until his condition improved. He died at 3 months of age.

### 2.2 Bioinformatic analyses of the novel c. 155t>C variant in *RRM2B*

Hierarchical bioinformatic analyses were performed after whole-exome sequencing (WES) of the patient's family. WES Genomic DNA was extracted from peripheral blood samples. WES kit (MyGenostics, Beijing) was used to capture the target, according to the manufacturer's instructions, and DNBSEQ-T7RS (MGI, China) sequenced the DNA. The pathogenicity of the variation was evaluated according to ACMG guidelines (22). The patient was homozygous for the NM_015713.5 (*RRM2B*): c.155T>C (chr8:103244426); NP_056528.2: p.Ile52Thr novel non-synonymous variant. The substitution at c.155 in exon 2 (c.155T>C) led to an amino acid change from isoleucine to threonine (p.Ile52Thr), which was confirmed by Sanger sequencing. In accordance with the sequencing results from the family, the patient's two copies of the variant were inherited from his parents, who were both heterozygous for this variant (Figures 2B,C). In contrast to their child, who had severe symptoms, the parents appeared to be asymptomatic. We found no record of this variant in the HGMD database or any suggestion of its pathogenicity in the latest version of the ClinVar database. In accordance with the ACMG standardized variant classification (https://www.acmg.net/docs/Standards_Guidelines_for_the_Interpretation_of_Sequence_Variants), the classification of the *RRM2B* (NM_015713.5): c.155T>C variant was “Unknown Significance”. This variant was preliminarily classified as a PP3_Strong + PM2_Supporting + PM3_ Supporting(hom) criteria, which means that its pathogenicity was supported by *in silico* evidence and the variant was absent from controls. No information about this site was found in the general population databases, which included gnomAD. The REVEL integrative functional prediction algorithm for non–synonymous variants was applied and the result was indicative of a harmful consequence. Orthodox ratings for functionality consistently gave a biologically harmful result from SIFT, PolyPhen_2, Mutation Taster and GERP++, based on the evolutionary conservation. The analysis of conservation showed that the amino acids at this site were highly conserved across multiple species, from fish to mammals (Figure 2D).

As a change in the primary protein structure was caused by this novel missense variant, AlphaFold2 and JPred4 were used to predict a potential alteration for the higher-order structure. In the protein structure model, a substitution of p.I52T in the small p53 inducible subunit (p53R2) led to altered physical and chemical characteristics, such as polarity, charge and isoelectric point (PI). The nonpolar, hydrophobic amino acid at position 52 changed to a polar, hydrophilic amino acid and the PI declined from 6.02 to 5.6. The potential role of the identified *RRM2B* variant was assessed by analyzing the p53R2 protein (encoded by *RRM2B*) structure generated by the AlphaFold artificial intelligence algorithm (Figure 1B). AlphaFold2 produced a per-residue confidence score >90, which indicated very high confidence in the model for most regions of the protein structure. The variant occurred in an alpha helix structure and resulted in conformation distortions that affected protein interactions. The protein secondary structure prediction by JPred4 showed a change in the alpha–helix in the region between amino acids 50 and 60, together with multiple other regions in the variant protein, when compared with the wild type. This implied that functionalization of the protein was affected during polymerization (Supplementary Figure S3). These results all suggested that c. 155T>C (p.I52T) had a high impact on protein function. Because infantile-onset *RRM2B*-related MDDS is an autosomal recessive disease, we proposed to change the original classification of “Unknown Significance” variant to “Likely Pathogenic” variant.

## 3 Previously reported pathogenic *RRM2B* variants

**Figure 3:**
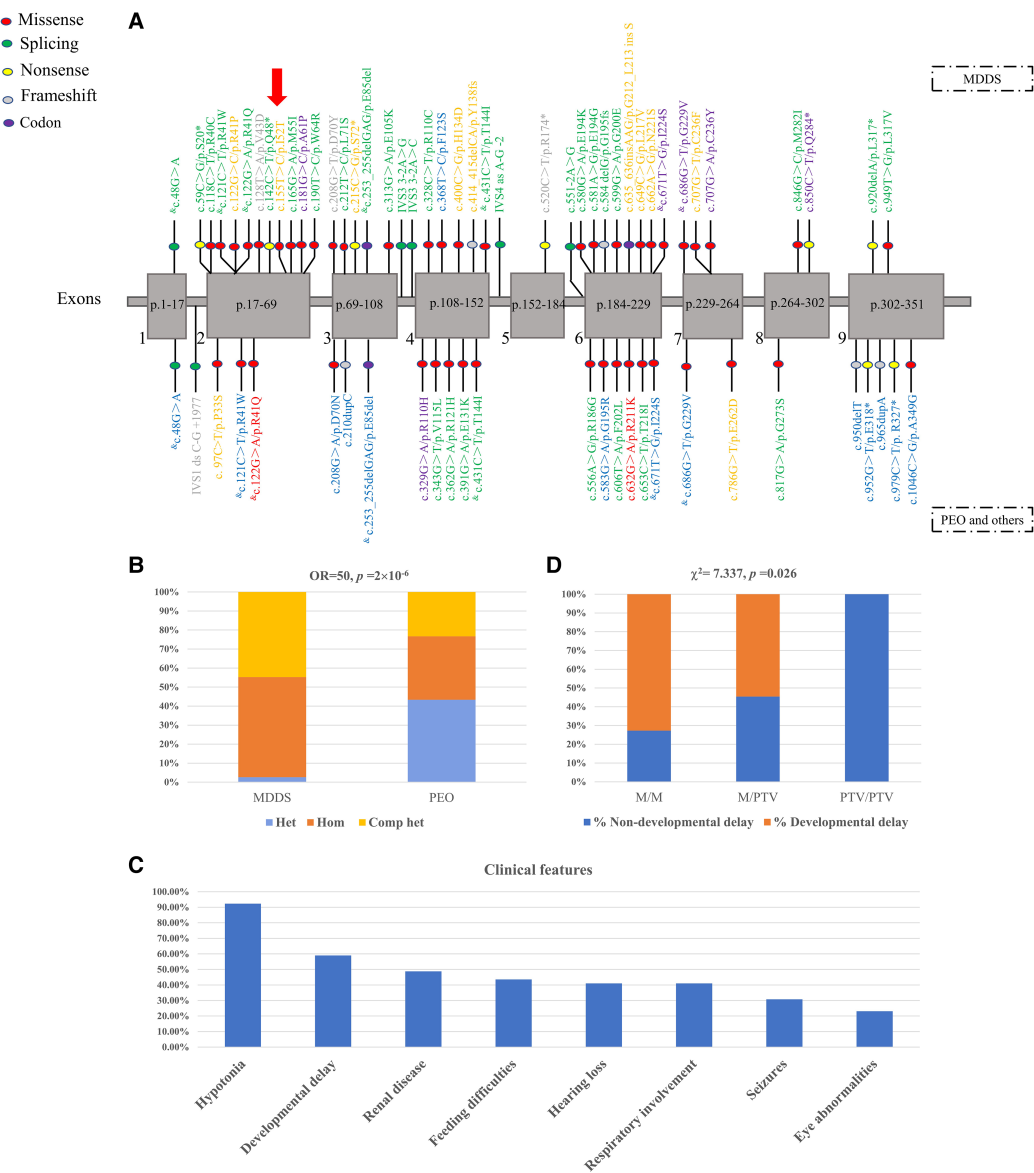
(**A**) previously reported pathogenic *RRM2B* variants and the novel variant in the patient. The location of each variant is shown in the nine exons of *RRM2B* messenger RNA (mRNA). The P53R2 protein comprises 351 amino acid residues. Each exon is labeled with the segment of the amino acid chain that it encodes. Codon variant refers to an insertion or deletion of one or several codons. The yellow, blue and green fonts represent homozygous, heterozygous and compound heterozygous genotypes, respectively. The grey font indicates an unknown type of variant. Purple represents both homozygous and compound heterozygous genotypes. Red represents both heterozygous and complex heterozygous genotypes. & indicates that the variant was present in both MDDS and PEO. The red arrow represents the novel variant. (**B**) Distribution of genotypes in patients with MDDS and PEO. M, missense variant; PTV, protein-truncating variant, e.g., splice-site, nonsense, frameshift and codon variants. (**C**) The relationship between PTV and developmental delay in patients with MDDS. (**D**) Clinical features in patients with MDDS.

We collated data on 63 variants from affected individuals with different clinical manifestations and of different ancestry to gain more insightful knowledge about the contribution to disease from variants in the *RRM2B* gene (Figure 3A, Supplementary Table S1). The reported pathogenic variants mainly affected the primary protein structure and included 41 missense variants (65.08%), eight nonsense variants (12.70%), six splicing variants (9.52%), seven frameshift variants (11.11%) and one large deletion (1.59%) from exon 4 to exon 6. The frameshift variants included a deletion (c.414_415delCA) and an insertion (c.635_636insAAG) of one codon. An uneven distribution of pathogenic variants was observed across each exon, with the highest density in exons 4, 6 and 2. The novel variant detected in this study was found in exon 2.

The genotype and the severity of the clinical manifestation was tightly correlated. The affected individuals were mainly diagnosed into two categories of diseases, MDDS and PEO, based on the clinical data (Supplementary Table S2). The variants found in patients with MDDS were mostly distributed in exons 2, 4 and 6, which was consistent with the overall trend, while the variants detected in patients with PEO were found in exons 4, 6 and 9 (Figure 3A, Supplementary Table S3). Single heterozygotes in PEO and homozygote or compound heterozygotes in MDDS were mostly found in exons 1, 3 and 9 (Figure 3A). The predominant variant types that were responsible for such diseases were the variants that altered the amino acids. These included 27 (64.29%) missense and four (9.52%) frameshift variants in patients with MDDS, and 19 (70.37%) missense and four (14.81%) frameshift variants in patients with PEO or other diseases. Among the reported variants, most were found separately in MDDS or PEO, with just seven shared between both diseases. Notably, the genotype distribution was significantly different across the two disease categories, with a striking enrichment of pathogenic variants in MDDS cases (Fisher's exact test, OR* *= 50, *p *= 2′10^−6^, Figure 3B). For MDDS, almost all variants were found in homozygous or compound heterozygous genotypes, which may indicate that both copies of the gene were damaged. In contrast, the genotypes were mainly heterozygous or compound heterozygous in patients with PEO, which suggested a partially disrupted function of the gene (Figure 3A; Supplementary Table S1).

## 4 The natural history of disorders with defects in *RRM2B*

We collated a detailed set of clinical data from the literature from 84 patients with a defect in *RRM2B* (Supplementary Table S2). The data was composed of 39 patients with MDDS, 36 patients with PEO, four individuals with renal dysfunction, rod-cone dystrophy and SNHL, two family members with acute liver failure and three patients without specific records of clinical symptoms. The patients with MDDS mostly developed severe symptoms that were lethal in infancy. However, the patients with PEO typically started to show symptoms on reaching maturity and the disease was not lethal.

Detailed clinical, biochemical and imaging features of all 39 pediatric patients with MDDS are summarized in Supplementary Table S2. The median age of onset was two months, with a range from 0 to 36 months, and 33 patients (84.62%) died. The most frequent clinical symptoms, such as hypotonia (36/39, 92.31%), developmental delay (23/39, 58.97%), renal disease (19/39, 48.72%), feeding difficulties (17/39, 43.59%), hearing loss (17/39, 43.59%), respiratory involvement (16/39, 41.03%), seizures (12/39, 30.77%) and eye abnormalities (9/39, 23.08%) (Figure 3C), involved multiple systems. Muscle residual mtDNA and plasma lactic acid were measured in 56.41% (22/39) and 66.67% (26/39) of patients, respectively, with 63.64% (14/22) of patients having significantly diminished mtDNA and all patients having significantly elevated plasma lactic acid. We found CSF protein or lactate in 35.9% (14/39) of patients and CK was found in 30.77% (12/39) of patients, with elevated levels in 71.43% (10/14) and 75% (9/12), respectively. An MRI scan was performed on 25.64% (10/39) of patients and 70% (7/10) were abnormal.

We conducted additional analyses using the collected MDDS data. A close relationship was found between both the age of onset and time of death and the residual mtDNA levels in skeletal muscle. A lower residual mtDNA level was associated with an earlier onset (Spearman's rank test, rho = 0.59, *p *= 0.034) and an earlier death (Spearman's rank test, rho = 0.676, *p *= 0.011). An earlier onset tended to result in an earlier death (Spearman's rank test, rs = 0.686, *p *= 0.01). Patients who presented with eye abnormalities tended to develop the syndrome later (Mann-Whitney U test, *p *= 0.049), while patients who showed developmental delay tended to die later (Mann-Whitney U test, *p *= 0.014). As shown in Figure 3C, the number of PTVs (protein-truncating variants), such as splice-sites, nonsense, frameshift and codon variants, was positively associated with a higher risk of developmental delay (Chi squared test, χ^2^* *= 7.337, *p *= 0.026).

## 5 Discussion

The high mortality rate of infantile MDDS and the lack of effective treatments lead to an extremely poor prognosis for patients with the disease, despite its extremely low morbidity. Progression of the disease is usually faster in patients with infantile MDDS caused by *RRM2B* defects and death comes at an earlier stage (1, 23). The common clinical manifestations are feeding difficulties, failure to thrive, hypotonia in newborns, respiratory distress and lactic acidosis, occasionally found with proximal tubulopathy, seizures, SNHL and GI symptoms (24, 25). Fewer than 40 cases of infantile-onset MDDS, caused by an *RRM2B* defect, have been reported and most developed lethal symptoms in infancy, with approximately 85% mortality (9, 18–21). A lack of etiological and pathogenic understanding of the disease remains a fundamental reason for the poor results from clinical intervention. It is necessary to carefully investigate each clinical case to raise the awareness of medical practitioners in discerning and diagnosing the syndrome and to promote the development of appropriate treatments for each affected patient.

In this study, we reported a patient whose *RRM2B* function was most likely impaired by a homozygous nonsynonymous variant. The patient was affected by the typical features of infantile MDDS, with respiratory distress and failure as his major cause of death. His parents refused permission for a muscle biopsy, which meant that residual muscle mtDNA could not be investigated. The novel variant in *RRM2B* was confirmed in the trio-family and was evaluated to be deleterious, based on its biological pathogenicity, through a set of functional predictions. It was considered to be a plausible cause of MDDS in the patient. The novel variant was found in a phylogenetically conserved site and may have a high impact on gene function. This further added to the evidence that this novel mutation is Likely Pathogenic. A comprehensive analysis of the clinical phenotype of the patient adds support for a correlation between genotype and pathogenesis of the disease.

The p53R2 protein that is encoded by *RRM2B* is a homolog of subunit R2 of RNR, which is a heterotetramer composed of two R1 subunits and two R2 subunits (8, 26). In the cytosol (3), RNR is essential for keeping an intact mitochondrial deoxyribonucleotides triphosphate (dNTP) pool and is required for the conversion of ribonucleotide diphosphate to deoxyribonucleotide diphosphate. Damage to p53R2 synthesis prevents the dNTP pool from being maintained for mtDNA replication, which leads to mtDNA depletion (Figure 1A). The *RRM2B* gene is widely expressed across human tissues, with an exceptional level of p53R2 found in skeletal muscle. The p53R2 protein takes over from R2 in tissues with a low amount of R2 and it plays a role in dNTP synthesis (27). Therefore, variants in *RRM2B* that reduce the levels or activity of p53R2 create an imbalanced dNTP pool, create a deficiency in mtDNA synthesis and ultimately cause MDDS. The newly discovered p.Ile52Thr substitution is close to the N-terminal of p53R2 and shows a high degree of conservation across species (Figure 2D). This suggested that the altered amino acid may impact the structure and function of p53R2 (Figure 1B). Moreover, this variant, at the 52nd position in the polypeptide, was very close to the p.M55I (c.165G>A) variant, which was previously reported to be pathogenic (18). The proximity of these two variants supported a common influence on the structure and activity of the polypeptide.

Our compiled data (Figure 3C, Supplementary Table S2) indicated notable phenotypic heterogeneity among the *RRM2B*-related diseases. The clinical phenotypes of other child- and adult-onset diseases caused by a defect in *RRM2B* (13), such as common PEO, ptosis, proximal muscle weakness, uncommon encephalopathy, GI and renal involvement, were distinct from infantile MDDS. We found that hearing loss occurred at a different frequency in infantile-onset MDDS (43.59%), when compared to other child or adult disorders (30.95%). This contrasts to the similar percentage of 36% observed in these two groups by Keshavan et al. (18), using a smaller data set. Seizures were observed in 30.77% of infantile MDDS cases in our data, which was also different to the 39% observed by Keshavan et al. (18). The phenotypic heterogeneity observed in our study resulted from a disparity in the spectrum of *RRM2B* variants between infantile-onset MDDS and other child or adult disorders.

The detailed analyses in this study showed a significant correlation between genotypes and phenotypic severity. Autosomal recessive inheritance was observed more often in the infantile-onset disorders than in other disorders, which were caused by recessive or dominant inheritance of pathogenic variants (13). The patients with MDDS with recessively inherited homozygote or compound heterozygote genotypes had a more severe multisystem condition and an earlier disease onset. Most died in infancy. In contrast, the symptoms of patients with single heterozygote variants emerged at a later age and were not as life-threatening. Notably, the biallelic *RRM2B* variants in more seriously affected infantile patients exhibited a departure in their distribution along the gene from those in other child or adult patients. The variable severity of the disease phenotypes may be attributable to the different functional domains in which the variants are found. Variants were mainly found in exons 2, 3, 4 and 6, in patients with infantile MDDS, and in exons 4, 6 and 9, in patients with PEO (Supplementary Table S3). Hence, it appeared that the variant position and genotype may exert different effects on the gene function implicated in the different diseases.

In addition to investigating the spectrum of infantile MDDS due to defects in *RRM2B*, we also attempted to expand our understanding of the correlation between different phenotypes. It has been found that the residual mtDNA levels reflect the RNR activity and seem to be related to certain clinical features, such as early death and age of onset, with marginal significance (13). Thus, the degree of mtDNA depletion can be used to predict the age of onset or the severity of the disease, in that a lower residual mtDNA level is associated with an earlier onset, an earlier death and a worse prognosis.

## 6 Conclusions

In summary, the novel c.155T>C variant in *RRM2B* that was identified in this study may be a genetic cause of MDDS and expands the variant spectrum of the *RRM2B* gene. The clinical characterization for the case should be used to raise the awareness of medical practitioners in discerning and diagnosing the syndrome and may promote the development of appropriate treatments for affected patients. We investigated the history of disorders found in combination with an *RRM2B* defect to improve our understanding of the genetic contribution to the disease spectrum. In previously reported cases of infantile MDDS there was a lack of investigation into the underlying molecular mechanisms of the disease. This study provided data to inspire future work to explore the functionality and pathogenicity of *RRM2B* variants in various disorders.

## Data Availability

The original contributions presented in the study are included in the article/**Supplementary Material**, further inquiries can be directed to the corresponding authors.
