# Supplementary material for: Case Report: A novel RRM2B variant in a Chinese infant with mitochondrial DNA depletion syndrome and collective analyses of RRM2B variants for disease etiology
Source: Front Pediatr. 2024 Apr 25;12:1363728. doi: 10.3389/fped.2024.1363728 (PMC11084280; doi:10.3389/fped.2024.1363728)
Supplement: Supplementary file 1 [file Datasheet1.pdf]

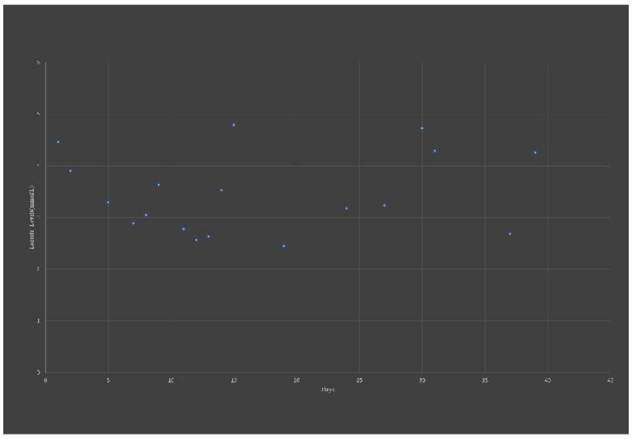

Supplementary Figure1. Daily plasma lactate levels for the patient from admission. The result showed that the patient had aberrantly elevated lactate all the time during the hospitalization.

Urine organic acid test results

Items	Reference range (μmol/L)	Results	Trends
Lactate -2	0-4.7	458.91	↑
Glycollic acid-2	0-2.2	13.21	↑
Oxalic acid-2	0	36.81	↑
2-hydroxybutyrate-2	0	102.07	↑
3-hydracrylicacid-2	0-1.1	11.32	↑
Pyruvic acid-OX-2	0-24.1	212.26	↑
3-hydroxybutyrate-2	0-3.7	1190.8	↑
3-hydroxyisobutyrate-2	0-9	48.06	↑
2-hydroxyisovalerale-2	0	30.73	↑
2-keto-isovaleric acid-OX-2	0-0.1	11.07	↑
Hydroxy propionate-2	0-2.9	16.9	↑
3-hydroxyvaleric acid-2	0	4.56	↑
Acetoacetic acid-OX-2	0	13	↑
2-keto-3-methylpentanoic acid-OX-2	0	15.31	↑
2-methyl-3-hydroxyvaleric acid-2(1)	0	7.75	↑
Glyceric acid-3	0-0.8	79.97	↑
Phosphoric acid -3	0-43	340.49	↑
2-methyl-3-hydroxyvaleric acid-2(2)	0	7.75	↑
2-keto-Isocaproic acid-OX-2	0	20.79	↑
Acetylglycine-1	0-0.1	3.48	↑
Maleic acid-2	0-0.4	1.94	↑
Fumaric acid-2	0-7.3	17.33	↑
Propionyl glycine-1	0	1.16	↑
2-deoxidize-4hydroxyacetoacetic acid	0-6.3	13.88	↑
3-methylpentenoic acid-2	0-4.2	7.16	↑
Butyrylglycine-2	0-0.7	0.68	↑
Malic acid-3	0-0.7	7.08	↑
5-oxyproline-2	0-7.6	85.16	↑
Thiodiglycolic acid-2	0	5.61	↑
7-hydroxyl-caprylic acid-2	0	0.66	↑
5-hydroxyl-methyl-2-furoic acid-1	0	11.2	↑
2-hydroxyglutaric acid-3	0.6-5.9	10.96	↑
3-hydroxyglutaric acid-3	0	19.95	↑
2-hydroxyadipic acid-3	0-2	4.7	↑
Vanillic acid	0	11.52	↑
Homovanillic acid-2	5.8-24.9	155.24	↑
Homogentisic acid-3	0-1.4	1.52	↑
4-hydroxyphenyllactic acid-2	0-7	97.68	↑
4-hydroxyphenylpyruvic acid-OX-2	0-0.9	2.12	↑
Palmitic acid-1	0-13.8	86.06	↑
3-hydroxycapric acid-3	0-4.4	16.18	↑
N-acetyltyrosine-3	0	0.57	↑
3-hydroxyl-dodecanedioic acid-3	0-1.4	3.09	↑

Supplementary Figure2. Urine organic acid test results. The result showed that a variety of urinary organic acids, such as lactate, pyruvic acid, hydroxybutyrate and 5-oxyproline, were elevated by varying degrees.

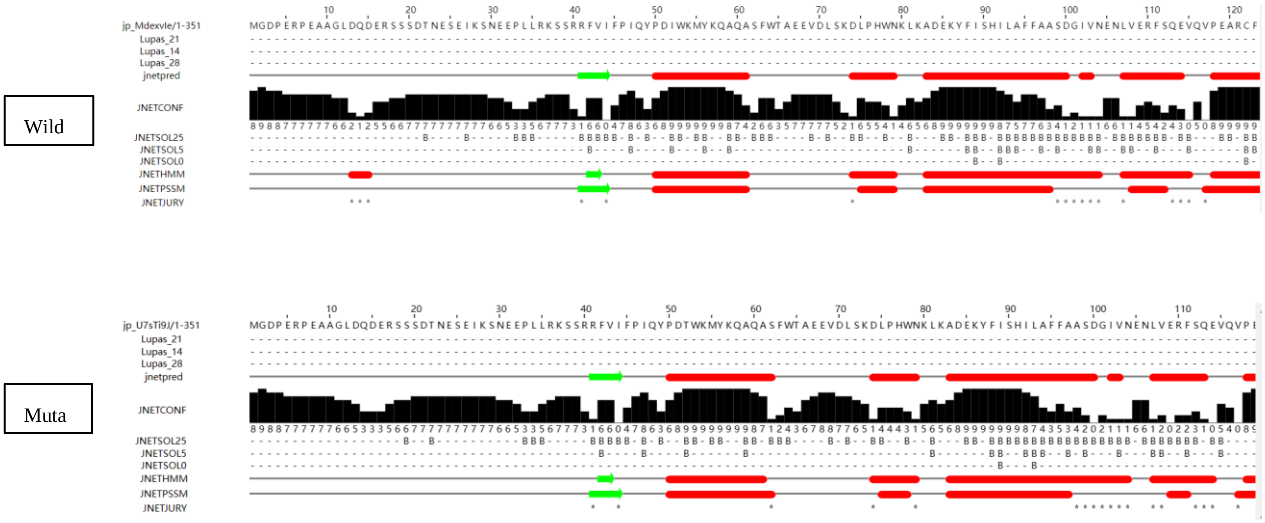

Supplementary Figure3. The amino acid change (p.I52T) in RRM2B resulted in a slight change in the alpha-helix, between amino acids 50 and 60. The red region is the alpha-helix and the green region is the β-strand.

Table S1. Variants in *RRM2B*

Number	Loci	Nucleotide	rs ID	MAF	Distribution	ACMG classification
1	Exon2	c.59C>G	chr8:103244522C>G	/	Infant	Pathogenic
2	Exon2	c.118C>T	rs776184830	0.000000	Infant	Likely pathogenic
3	Exon2	c.122G>C	rs200273673	0.000000	Infant	Pathogenic
4	Exon2	c.128T>A	rs1587186073	/	Infant	Pathogenic
5	**Exon2**	**c.155T>C**	**chr8:103244426T>C**	**/**	**Infant**	Likely pathogenic
6	Exon2	c.165G>A	chr8:103244416G>A	/	Infant	Likely pathogenic
7	Exon2	c.181G>C	chr8:103244400G>C	/	Infant	Uncertain
8	Exon2	c.190T>C	rs515726182	/	Infant	Pathogenic
9	Exon3	c.215C>G	chr8:103238252C>G	/	Infant	Pathogenic
10	Exon3	c.313G>A	chr8:103238154	/	Infant	Likely pathogenic
11	Intron3	IVS3 3–2A>G	rs515726185	/	Infant	Pathogenic
12	Intron3	IVS3 3–2A>C	rs515726185	/	Infant	Pathogenic
13	Exon3	c.212T>C	chr8:103238255T>C	/	Infant	Pathogenic
14	Exon4	c.328C>T	rs515726186	0.000007	Infant	Pathogenic
15	Exon4	c.368T>C	rs515726187	0.000004	Infant	Uncertain
16	Exon4	c.400C>G	chr8:103237168C>G	/	Infant	Pathogenic
17	Exon4	c.414_415delCA	103237152-103237154delCA	/	Infant	Pathogenic
18	Intron4	IVS4 as A-G -2	chr8:103236370	/	Infant	Pathogenic
19	Intron5	c.551-2A＞G	chr8:103231177	/	Infant	Pathogenic
20	Exon6	c.580G>A	rs121918308	0.000008	Infant	Pathogenic
21	Exon6	c.581A>G	rs515726191	/	Infant	Pathogenic
22	Exon6	c.584delG	chr8:103231141-103231142delG	/	Infant	Pathogenic
23	Exon6	c.599G>A	rs863224192	/	Infant	Pathogenic
24	Exon6	c.635_636insAAG	chr8:103231090insAAG	/	Infant	Likely pathogenic
25	Exon6	c.649C>G	rs755176920	0.000004	Infant	Likely pathogenic
26	Exon6	c.662A>G	rs863224193	0.000004	Infant	Pathogenic
27	Exon7	c.707G>A	rs121918309	0.000000	Infant	Pathogenic
28	Exon7	c.707G>T	rs121918309	0.000000	Infant	Pathogenic
29	Exon8	c.846G>C	rs182614164	0.000050	Infant	Uncertain
30	Exon8	c.850C>T	rs121918307	0.000004	Infant	Pathogenic
31	Exon9	c.920delA	chr8:103220496-103220497delA	/	Infant	Pathogenic
32	Exon9	c.949T>G	rs515726198	/	Infant	Likely pathogenic
33	Exon 4–6 deletion	c.(321+1_322–1)_(684+1_685-1)del	/	/	Infant	/
34	Exon1	c.48G>A	rs515726180	0.000021	Infant/aldult	Likely pathogenic
35	Exon2	c.121C>T	rs515726181	0.000000	Infant/aldult	Pathogenic
36	Exon2	c.122G>A	rs200273673	0.000000	Infant/aldult	Likely pathogenic
37	Exon3	c.253_255delGAG	rs515726184	0.000007	Infant/aldult	Likely pathogenic
38	Exon6	c.671T>G	rs515726196	0.000029	Infant/adult	Pathogenic
39	Exon6	c.632G>A	rs515726195	0.000012	infant /adult	Likely pathogenic
40	Exon7	c.686G>T	rs121918311	0.000016	Infant/teenager	Pathogenic
41	Exon2	c.142C>T	chr8:103244439C>T	/	Pediatric patien	Pathogenic
42	Exon3	c.210dupC	chr8:103238256dupC	/	Teenager	Pathogenic
43	Exon4	c.431C>T	rs515726189	0.000004	Pediatric patient/ infant/ teenager	Uncertain
44	Exon6	c.556A>G	rs515726190	0.000004	Teenager	Pathogenic
45	Exon6	c.606T>A	rs515726194	/	Preschooler	Uncertain
46	Exon6	c.653C>T	chr8:103231073	/	Teenager	Likely pathogenic
47	Exon7	c.786G>T	rs1810682433	/	Preschooler	Likely pathogenic
48	Exon8	c.817G>A	rs387906891	0.000024	Preschooler	Uncertain
49	Exon2	c.97C>T	rs387906892	0.000008	Adult	Uncertain
50	Exon3	c.208G>A	rs515726183	0.000000	Adult	Likely pathogenic
51	Exon4	c.329G>A	rs515726186	0.000070	Adult	Pathogenic
52	Exon4	c.343G>T	chr8:103237225G>T	/	Adult	Uncertain
53	Exon4	c.362G>A	rs267607024	0.000004	Adult	Pathogenic
54	Exon4	c.391G>A	rs515726188	0.000018	Adult	Uncertain
55	Exon6	c.583G>A	rs515726192	0.000004	Adult	Uncertain
56	Exon9	c.950delT	chr8:103220466delT	/	Adult	Pathogenic
57	Exon9	c.952G>T	rs515726200	/	Adult	Likely pathogenic
58	Exon9	c.965dupA	chr8:103220451	/	Adult	Likely pathogenic
59	Exon9	c.979C>T	rs121918310	0.000000	Adult	Likely pathogenic
60	Exon9	c.1046C>G	rs515726202	/	Adult	Likely pathogenic
61	Intron1	IVS1 ds C-G +1977	chr8:103249078	/	/	/
62	Exon3	c.208G>T	rs515726183	0.000000	/	Pathogenic
63	Exon5	c.520C>T	rs863224191	0.000100	/	Pathogenic

Note: MAF, Minor Allele frequency in genomAD. Novel mutation is highlighted in bold. Gene reference sequence: GRch37, NM_015713.5; Protein reference sequence: NP_056528.2.

Table S2. Clinical and genetic characteristics of reported cases with RRM2B variants

Reference (Pubmed ID)	Variants in each case	ACMG classification	Zygosity	Gender	Age onset	Outcome	Origin	Phenotype	Muscle mtDNA (residual amount)	Serum lactate (＜2.2mmol/L)	CSF protein (0-0.4g/L)/ lactate (0.9-	MRI	CK
Riley et al, 2020 (32313153)	c.48G>A and c.118C>T	Likely pathogenic/Likely pathogenic	Comp het	Female	3y	Death at 10y	Australia	Global developmental delay, deafness, retinopathy, renal failure, cognitive decline, seizures	NA	NA	NA/2.5	Widespread extensive white matter changes especially in the deep frontal lobes	Rise
Fang et al, 2017 (28639102)	c.121C>T and c.122G>A	Pathogenic/Likely pathogenic	Comp het	Male	4m	NA	China	Hypotonia, dystonia, muscle weakness, muscularatrophy, feeding difficulties, failure to	NA	NA	NA	NA	NA
Spinazzola et al, 2009 (19125351)	c.122G>C	Pathogenic	Hom	Female	Neonate	Death at 4.5m	Italy	Floppiness, congenital deafness, glycosuria	5%	Significant rise	NA	NA	Rise
	IVS3 3–2A>C and c.328C>T	Pathogenic/Pathogenic	Comp het	Male	2m	Death at 12m	Italy	Floppiness, tubulopathy	10%	Significant rise	NA	NA	NA
**This study**	c.155T>C	Likely pathogenic	Hom	Male	2m	Death at 3m	China	Hypotonia, lactic acidosis,feeding difficulties	NA	2.4-4.7	1.94/NA	Water content of white matter of bilateral frontal, temporal, parietal and occipital lobes was evaluated. The sulci of bilateral cerebral hemispheres were widened and deepened	Rise
Bourdon et al,2007 (17486094)	c.850C>T	Pathogenic	Hom	Female	1m	Death at 2m	Moroccan	Hypotonia, lactic acidosis, proximal tubulopathy	1-2%	5.5	NA	NA	NA
	c.850C>T	Pathogenic	Hom	Female	1m	Death at 2m		Hypotonia, lactic acidosis, proximal tubulopathy	NA	NA	NA	NA	NA
	c.850C>T	Pathogenic	Hom	Male	1m	Death at 2m		Hypotonia, lactic acidosis, proximal tubulopathy	1%	NA	NA	NA	NA
	c.850C>T IVS3 3–2A>G and c.580G>A	Pathogenic/Pathogenic/Pathogenic	Comp het	Male	Neonate	Death at 2m	French	Hypotonia, tubulopathy, seizures	2%	6.5	NA/3.3	NA	NA
	c.850C>T IVS3 3–2A>G and c.580G>A	Pathogenic/Pathogenic/Pathogenic	Comp het	Female	Neonate	Death at 2m		Neonatal hyperlactatemia, tubulopathy, seizures	NA	NA	NA	NA	NA
	c.850C>T c.190T>C and c.581A>G	Pathogenic/Pathogenic/Pathogenic	Comp het	Female	Neonate	Death at 4m	French	Hypotonia, respiratory distress, hyperlactatemia, tubulopathy	1-2%	NA	NA	NA	NA
	c.850C>T c.253_255delGAG and c.707G>T	Pathogenic/Likely pathogenic/Pathogenic	Comp het	Female	Neonate	Death at 3m	French	Trunk hypotonia, vomiting and diarrhea, hyperlactatemia	1%	6.79	NA/3.5	NA	NA
Kropach et al,2017 (28482374)	c.215C>G	Pathogenic	Hom	Male	2.5m	Death at 3m	Arab Muslim	Lethargic, with a high-pitched cry, severe hypotonia, patellar areflexia, dysmorphic features, focal seizures, respiratory failure	NA	20-48	0.3/NA	Normal	Rise
	c.215C>G	Pathogenic	Hom	Male	1m	Death at 2.5m		Hypotonia, deafness, tubular dysfunction, respiratory failure	NA	60-120	0.16/NA	Normal	NA
Acham-Roschitz et al, 2009 (19616983)	c.368T>C and SNP rs72554091 (promoter)	Uncertain/NA	Het	Female	2m	Death at 4m	Australia	Lack of head control, truncal hypotonia, nephrocalcinosis, proximal tubulopathy, vomiting, respiratory distress, focal seizures	4%	1.3-8.9	NA	No lactate peak was detected on [1] H-MRS	Normal
Pronicka et al , 2016 (27290639)	c.414_415delCA	Pathogenic	Hom	Female	Neonate	Death at 4m	Poland	Deafness, hypotony, neurological regression, lactic acidosis, mtDNA depletion in muscle	＜30%	NA	NA	NA	NA
	c.686G>T	Pathogenic	Hom	Male	Neonate	Death at 2y	Poland	Encephalopathy, cataract, cardiomyopathy, lactate, branched chain amioacids, mtDNA	＜30%	NA	NA	NA	NA
Fumagalli et al, 2022 (35756861)	c.551-2A > G and c.686G>T	Pathogenic/Pathogenic	Comp het	Female	Neonate	Death at 2m	Italy	Poor suck, mild hypotonia, lethargy, glycosuria and proteinuria, metabolic acidosis with fluctuating hyperlactacidemia, sensorineural	NA	2.0-12.0	NA	Diffuse white matter hyper-intensity on T2-weighted imaging, reduced subarachnoid spaces and slit-like ventricles. No lactate peak was	NA
	c.671T>G	Pathogenic	Hom	Female	2m	Death at 8m	USA	Watery diarrhea, persistent acidosis, progressive weakness, poor head control, worsening respiratory, central sensorineural hearing loss	< 2%	2.8-17.4	0.164/5.2	NA	NA
Bornstein et al, 2008 (18504129)	c.846G>C and c.920 delA	Uncertain/Pathogenic	Comp het	Male	4 m	At least 4y	USA	Encephalomyopathy, with microcephaly and global developmental delay	7.60%	4.3	a lactate peak	Bilateral and nearly symmetrical non-enhancing areas of abnormal signal and reduced diffusion in the white matter. [1] H-MRS showed a lactate peak	Normal
	c.584delG and c.949T>G	Pathogenic/Likely pathogenic	Comp het	Female	6m	At least 27m	Spain	Progressively worsening hypotonia, failure to thrive, microcephaly	11.00%	4.1-7.2	NA	NA	Rise
Bychkov et al, 2021 (22486010)	c.649C>G	Likely pathogenic	Hom	NA	Neonate	Death at 3m	Russia	Severe multi-organ disease with renal failure, hepatic insufficiency, and hypertrophic	NA	NA	NA	NA	NA
Penque et al, 2018 (30439532)	c.662A>G	Pathogenic	Hom	Male	2m	Death at 3m	Latino	Progressive hypotonia, failure to thrive, severe lactic acidosis, deafness	NA	3.0-28.0	NA	Normal	Rise
Kollberg et al, 2009 (19138848)	c.686G>T	Pathogenic	Hom	Male	2m	Death at 3m	Sudan	Feeding difficulties with failure to thrive, muscular hypotonia, lactic acidosis, renal proximal tubulopathy, poor visual contact,	3-4%	NA	NA	NA	Normal
	c.686G>T	Pathogenic	Hom	Male	2m	Death at 5m		Tonic-clonic seizures, feeding difficulties with failure to thrive and progressive muscle weakness, hypotonia, lactic acidosis, poor visual contact	1-2%	9.1	1.16/7.0	Generalized atrophy	Rise
Stojanović et al, 2013 (24382854)	c.707G>A	Pathogenic	Hom	Male	3m	Death at 8m	Serbia	Myopathy, deafness, peripheral neuropathy, nephrocalcinosis, proximal renal tubulopathy	NA	2.0-8.0	NA	NA	Normal
E. J. Theunissen et al, 2018 (30369941)	c.142C>T and c.431C>T	Pathogenic/Uncertain	Comp het	NA	Pediatric	NA	Netherlands	Hearing problems, muscle hypotonia, mitochondrial encephalopathy, motor developmental delay,	NA	NA	NA	NA	NA
Dai et al, 2019 (30818899)	c.212T>C and IVS4 as A-G -2	Pathogenic/Pathogenic	Comp het	Female	Neonate	At least 4m	China	Feeding difficulties, developmental delay, muscle hypotonia, dysphagia, hearing problems	NA	5.3	NA	Symmetrically long T2 signals were found bilaterally in the heads of the putamen and caudate nuclei	Rise
Keshavan et al, 2020 (31462754)	c.599G＞A and c.313 G＞A	Pathogenic/Likely pathogenic	Comp het	NA	Within 2m	5 cases: death at 3-6m. 1 case: death at 11m. 1 case: death at 13m. 1 case: death at 22m	UK, USA, Frence	All 9 cases: Feeding difficulties, lack of head control, truncal hypotonis, respiratory failure. 8 cases: generalised weakness. 6 cases: discoordinated suck, loss of reflexes. 5 cases: seizures, discoordinated swallow, poor visual contact. 4 cases: tubulopathy	NA	7	NA	NA	Rise
	c.181G＞C	Uncertain	Hom						6%	4.6	NA/3.5	NA	Rise
	c.165G＞A and exon 4–6 deletion	Likely pathogenic/NA	Comp het						5%	3.2	NA	NA	Normal
	c.181G＞C and c.846G＞C	Uncertain/Uncertain	Comp het						20%	5	NA	NA	Normal
	c.635_636insAAG	Likely pathogenic	Hom						NA	5.7	NA/5.6	NA	Rise
	c.635_636insAAG	Likely pathogenic	Hom						4%	6.1	NA	NA	Normal
	c.121C>T and c.671T>G	Pathogenic/Pathogenic	Comp het						6%	8.2	NA/3.3	NA	Normal
	c.400C>T	Pathogenic	Hom						3.10%	7.4	NA/4.7	NA	Rise
	c.59C>G and c.48G>A	Pathogenic/Likely pathogenic	Comp het						2%	41.7	NA/2	NA	Normal
Clinvar database	c.128T＞A	Pathogenic	NA	NA	Infant	NA	China	Mitochondrial DNA depletion syndrome 8a	NA	NA	NA	NA	NA
	c.520C>T	Pathogenic	NA	NA	NA	NA	NA	NA	NA	NA	NA	NA	NA
	c.662A>G	Pathogenic	Hom	Female	4m	NA	NA	Renal tubular acidosis, congenital glaucoma, hypotonia, sensorineural hearing loss	NA	NA	NA	NA	NA
	c.208G>T	Likely pathogenic	NA	NA	NA	NA	NA	NA	NA	NA	NA	NA	NA
Fratter et al, 2011 (21646632)	c.122G>A	Likely pathogenic	Het	NA	59y	At least 71y	England	PEO, no ptosis, encephalopathy, stroke-like episodes, deaf, cognitive decline	NA	NA	NA	NA	NA
	c.583G>A	Uncertain	Het	NA	50y	At least 75y		PEO, ptosis, deaf, dysphonia, proximal myopathy, ataxia, cataracts, glaucoma	NA	NA	NA	NA	NA
	c.686G＞T	Pathogenic	Het	NA	15y	At least 25y		Severe PEO, ptosis, dysphagia, dysphonia, fatigue, ataxia, proximal myopathy, deaf, facial weakness, IBS	NA	NA	NA	NA	NA
	c.950delT	Pathogenic	Het	NA	30y	At least 52y		PEO, ptosis	NA	NA	NA	NA	NA
	c.950delT	Pathogenic	Het	NA	53y	At least 73y		Severe PEO, ptosis, mild dysphagia, fatigue, ataxia, glaucoma	NA	NA	NA	NA	NA
	c.950delT	Pathogenic	Het	NA	46y	At least 66y		Severe PEO, ptosis, proximal myopathy,ataxia	NA	NA	NA	NA	NA
	c.952G>T	Likely pathogenic	Het	NA	53y	At least 64y		PEO, ptosis, dysarthria, dysphagia, fatigue	NA	NA	NA	NA	NA
	c.965dupA	Likely pathogenic	Het	NA	30y	At least 62y		PEO, fatigue, diabetes, GI symptoms	NA	NA	NA	NA	NA
	c.965dupA	Likely pathogenic	Het	NA	54y	At least 64y		PEO, ptosis, dysphagia, fatigue	NA	NA	NA	NA	NA
	c.965dupA	Likely pathogenic	Het	NA	26y	At least 61y		PEO, ptosis, dysphagia, fatigue, proximal myopathy, dysphonia, irritable bowel syndrome	NA	NA	NA	NA	NA
	c.431C>T and c.632G>A	Uncertain/Likely pathogenic	Comp het	NA	Birth	Death at 25y		PEO, ptosis, myopathy, renal failure, deaf, developmental delay, hypoparathyroidism, hypogonadism, areflexia, dysphagia	NA	NA	NA	NA	NA
	c.606T>A and c.817G	Uncertain/Uncertain	Comp het	NA	4y	At least 14y		PEO, ptosis, deaf	NA	NA	NA	NA	NA
Pitceathly et al,2012 (23107649)	c.48G>A	Likely pathogenic	Het	NA	63y	At least 64y	England	Mild PEO, ptosis, proximalmuscle weakness, ataxia and SNHL	NA	NA	NA	NA	NA
	c.48G>A	Likely pathogenic	Het	Female	70y	At least 72y		Mild PEO, ptosis, proximal muscle weakness, ataxia, hypothyroidism and SNHL	NA	NA	NA	NA	NA
	c.48G>A	Likely pathogenic	Het	NA	51y	At least 66y		PEO, ptosis, headaches, tinnitus, stroke-like episodes, fatigue and renal disturbance (renal calculi)	NA	NA	NA	NA	NA
	c.121C>T	Pathogenic	Het	NA	60y	Death at 66y		PEO, ptosis, migraine, neck flexion weakness (mild) and proximal muscle weakness	NA	NA	NA	NA	NA
	c.208G>A	Likely pathogenic	Het	NA	38y	At least 64y		PEO, ptosis, ataxia, proximal muscle weakness, bulbar symptoms, facial weakness, IHD and sleep disorder	NA	NA	NA	NA	NA
	c.583G>A	Uncertain	Het	Male	40y	At least 48y		PEO, ptosis	NA	NA	NA	NA	NA
	c.632G＞A	Likely pathogenic	Het	NA	30y	At least 82y		PEO, ptosis	NA	NA	NA	NA	NA
	c.671T>G	Pathogenic	Het	NA	48y	At least 62y		Severe PEO, diplopia, proximal muscle,weakness, cataracts and migraine	NA	NA	NA	NA	NA
	c.965dupA	Likely pathogenic	Het	Female	60y	At least 74y		Severe PEO, ptosis, facial weakness, fatigue and respiratory failure	NA	NA	NA	NA	NA
	c.965dupA	Likely pathogenic	Het	NA	40y	At least 64y		PEO, ptosis, ataxia and dysphagia	NA	NA	NA	NA	NA
	c.979C＞T	Likely pathogenic	Het	NA	55y	At least 71y		Severe PEO, ptosis, diplopia, proximal muscle weakness, ataxia and fatigue	NA	NA	NA	NA	NA
	c.979C＞T	Likely pathogenic	Het	Female	43y	At least 48y		PEO, asymmetrical ptosis, ataxia and fatigue	NA	NA	NA	NA	NA
	c.1046C>G	Likely pathogenic	Het	NA	45y	At least 62y		PEO, ptosis, proximal muscle weak-ness, fatigue, tremor, impaired memory and breast carcinoma	NA	NA	NA	NA	NA
	c.431 C＞T and c.817G＞A	Uncertain/Uncertain	Comp het	NA	14y	At least 37y		Severe PEO, ptosis, proximal muscle weakness, facial weakness, scapulae winging, low BMI, hypogonadism and osteoporosis	NA	NA	NA	NA	NA
	c.556A＞G and c.653C＞T	Pathogenic/Likely pathogenic	Comp het	NA	11y	At least 43y		Severe PEO, asymmetrical ptosis,proximal and distal muscle weakness, ataxia, SNHL, facial weakness,low BMI, leukoencephalopathy and depression	NA	NA	NA	NA	NA
Pitceathly et al, 2011 (21378381)	c.122G>A and c.391G＞A	Pathogenic/Uncertain	Comp het	NA	Adult	NA	England	Kearns–Sayre syndrome	NA	NA	NA	NA	NA
	c.253_255delGAG	Likely pathogenic	Het	NA	Adult	NA		Late-onset progressive external ophthalmoplegia, fatigue	NA	NA	NA	NA	NA
Heighton et al, 2019 (31521625)	c.253_255delGAG	Likely pathogenic	Het	Male	62y	NA	Canada	Ophthalmoplegia, ptosis	NA	NA	NA	NA	NA
J. Bandele et al, 2011 (20817676)	IVS1 ds C-G +1977	NA	NA	NA	NA	NA	NA	NA	NA	NA	NA	NA	NA
Takata et al, 2011 (21951382)	c.97C>T	Uncertain	Hom	Male	43y	NA	Japan	Depression, anxiety, hypochondriac complaints, hilateral perceptive deafness, ptosis, PEO, muscle weakness, pigmentary degeneration of retina, gonadal atrophy	NA	2	NA	T2-weighted MRI showed diffuse hyper-intensity in the cerebral white matter, especially in the occipital periventricular regions	NA
Valencia et al, 2016 (27483465)	c.210dupC	Pathogenic	Het	Male	16y	At least 27y	Hispanic	Acute liver failure	＜30%	NA	NA	NA	NA
	c.210dupC	Pathogenic	Het	Male	3y	At age 7y	Caucasian	Reccurent acute liver failure	NA	NA	NA	NA	NA
Shaibani et al, 2009 (19667227)	c.329G>A and c.362G>A	Pathogenic/Pathogenic	Comp het	Female	42	At least 42y	America	Ophthalmoplegia, ptosis, gastrointestinal dysmotility, cachexia, peripheral neuropathy	12%	2.8	Both are slightly elevated	Increased T2-weighted signal in the basal ganglia and patchy T2-weighted signals throughout the periventricular and subcortical white matter	Normal
rodríguez- López et al, 2020 (32161153)	c.329G>A	Pathogenic	Hom	NA	NA	NA	Spain	PEO which associates extraocular symptoms	NA	NA	NA	NA	NA
	c.343G>T and c. 817G>A	Uncertain/Uncertain	Comp het	NA	NA	NA	Spain	Kearns- Sayre syndrome, PEO which associates extraocular symptoms	NA	NA	NA	NA	NA
Roberts et al, 2020 (32827185) and Beighton et al, 1993 (8279480)	c.786G>T	Likely pathogenic	Hom	Female	4y	Death at early adulthood	South Africa	Deafness, retinitis pigmentosa, Fanconi type of renal lesion, small stature, hearing loss	NA	NA	NA	NA	NA
	c.786G>T	Likely pathogenic	Hom	Female	1.5y	Death at early adulthood		Deafness, retinitis pigmentosa, Fanconi type of renal lesion, small stature, hearing loss	NA	NA	NA	NA	NA
Roberts et al, 2020 (32827185)	c.786G>T	Likely pathogenic	Hom	Male	2	At least 18y		Hearing loss, renal rickets, blindness, Fanconi-type renal disease and rod-cone dystrophy	NA	NA	NA	NA	NA
	c.786G>T	Likely pathogenic	Hom	Female	4	At least 13y		Hearing loss, renal rickets, Fanconi-type renal disease and rod-cone dystrophy	NA	NA	NA	NA	NA
Tyynismaa et al, 2009 (19664747)	c.979C>T	Likely pathogenic	Het	Male and female	NA	NA	Finland	PEO	about 70%	NA	NA	NA	NA
	c.979C>T	Likely pathogenic	Het	Male and female	NA	NA		PEO	about 70%	NA	NA	NA	NA

Comp het, compound heterozygous; Hom, homozygous; Het. heterozygous; m, month-old; y, years old; PEO, progressive external ophthalmoplegia; SNHL, sensorineural hearing loss; [1] H-MRS, proton magnetic resonance spectroscopy; NA,no data.

Table S3. Distribution of variants in different locations

Locid	Amino acid number of	Total		MDDS		PEO and others	
		Frequency	Percent	Frequency	Percent	Frequency	Percent
Exon1	17	1	5.88%	1	5.88%	1	5.88%
Exon2	53	12	22.64%	11	20.75%	3	5.66%
Exon3	40	7	17.50%	5	12.50%	3	7.50%
Exon4	45	10	22.22%	5	13.33%	5	11.11%
Exon5	33	1	3.03%	1	6.06%	0	0.00%
Exon6	46	13	28.26%	8	19.57%	6	13.04%
Exon7	36	4	11.11%	3	8.33%	2	5.56%
Exon8	39	3	7.69%	2	5.13%	1	2.56%
Exon9	50	7	14%	2	4.00%	5	10%
Intron1	/	1	/	0	/	1	/
Intron3	/	2	/	2	/	0	/
Intron4	/	1	/	1	/	0	/
Intron5	/	1	/	1	/	0	/
